# Recent Trends on Mitigative Effect of Probiotics on Oxidative-Stress-Induced Gut Dysfunction in Broilers under Necrotic Enteritis Challenge: A Review

**DOI:** 10.3390/antiox12040911

**Published:** 2023-04-11

**Authors:** Uchechukwu Edna Obianwuna, Nenna Agbai Kalu, Jing Wang, Haijun Zhang, Guanghai Qi, Kai Qiu, Shugeng Wu

**Affiliations:** 1 National Engineering Research Center of Biological Feed, Institute of Feed Research, Chinese Academy of Agricultural Sciences, Beijing 100081, China; 2 Department of Animal Science, Ahmadu Bello University, Zaria 810211, Nigeria

**Keywords:** gut health, broilers, oxidative stress, necrotic enteritis, probiotics

## Abstract

Gut health includes normal intestinal physiology, complete intestinal epithelial barrier, efficient immune response, sustained inflammatory balance, healthy microbiota, high nutrient absorption efficiency, nutrient metabolism, and energy balance. One of the diseases that causes severe economic losses to farmers is necrotic enteritis, which occurs primarily in the gut and is associated with high mortality rate. Necrotic enteritis (NE) primarily damages the intestinal mucosa, thereby inducing intestinal inflammation and high immune response which diverts nutrients and energy needed for growth to response mediated effects. In the era of antibiotic ban, dietary interventions like microbial therapy (probiotics) to reduce inflammation, paracellular permeability, and promote gut homeostasis may be the best way to reduce broiler production losses. The current review highlights the severity effects of NE; intestinal inflammation, gut lesions, alteration of gut microbiota balance, cell apoptosis, reduced growth performance, and death. These negative effects are consequences of; disrupted intestinal barrier function and villi development, altered expression of tight junction proteins and protein structure, increased translocation of endotoxins and excessive stimulation of proinflammatory cytokines. We further explored the mechanisms by which probiotics mitigate NE challenge and restore the gut integrity of birds under disease stress; synthesis of metabolites and bacteriocins, competitive exclusion of pathogens, upregulation of tight junction proteins and adhesion molecules, increased secretion of intestinal secretory immunoglobulins and enzymes, reduction in pro-inflammatory cytokines and immune response and the increased production of anti-inflammatory cytokines and immune boost via the modulation of the TLR/NF-ĸ pathway. Furthermore, increased beneficial microbes in the gut microbiome improve nutrient utilization, host immunity, and energy metabolism. Probiotics along with biosecurity measures could mitigate the adverse effects of NE in broiler production.

## 1. Introduction

The ever-growing increase in the poultry industry accounts for its key economic contribution to food security and human nutrition [1]. The global consumption of poultry meat and eggs compared to other source of proteins confers on it a comparative advantage. Broilers are often considered in poultry production due to their fast-growing nature, the nutrient absorption capacity of the intestinal epithelium and the high rate of nutrient conversion to muscle [2]. In the recent times, significant improvement has been made in the broiler breeding industry in a bid use genetic selection to achieve an increased feed conversion ratio, efficient conversion of feed nutrients to muscle, fat reduction and so on [3]. However, selection for high yield in the development of modern intensive broiler farming exposes the birds to oxidative stress, and broilers are prone to oxidative stress under certain unsuitable physiological and environmental conditions [4].

Oxidative stress, a common physiological process in life, is often characterized by the excessive production of several reactive oxygen species (hydroxyl free radicals and superoxide anions) in the body due to mitochondria’s oxidative phosphorylation reaction [5]. The accumulation of reactive oxygen species (ROS) causes the alteration of nucleic acid, biological macromolecules, lipid peroxidation evidenced by increased malondialdehyde content, damage of tissue and protein structures and, finally, cell apoptosis [6]. The gastrointestinal tract is primarily involved in nutrient absorption and immune regulation; it is protected by intestinal epithelia, regulatory molecules/proteins present on various epithelial cells, and connective tissues which are found on mucosal surfaces [7]. The activities of antioxidant enzymes and antioxidant genes, which constitute the gut antioxidant system, exert protective effects on the intestinal structure [8]. The exposure of the gastrointestinal tract (GIT) to oxidative stress accruing from several factors such as age, feed toxins, environmental stress and pathogens leads to the generation of ROS, which disrupts the redox balance and damages the intestinal structures, because the scavenging capacity of the antioxidant system is exceeded [9]. The susceptibility of the small intestine to oxidative stress is high, damaging the intestinal epithelium, which is the first line of defense [10]. Intestinal epithelium damage culminates in; pathogen invasion, toxin and endotoxin accumulation, molecular lesions that may cause apoptosis, tight junction protein disruption, and changes in villi morphology and microbiota composition [11,12,13]. Additionally, it diminishes the capacity of the antioxidant defense system, which is reflected in reduced antioxidant enzymes and genes, thereby reducing its resistance to disease [14]. Growth performance indices, including weight gain, feed intake and the feed conversion ratio, are the major determinants used to assess the economic returns in broiler production [15]. Most often, genetic selection for growth performance in modern-day broiler breeding may cause reduced disease resistance and natural immunity, hence the need for gut enhancers. Achieving efficient nutrient absorption and utilization for improved weight gain and muscle size in broiler production hinges on the utilization of gut enhancers to eliminate pathogen invasion and its negative effect, thus reducing oxidative stress in the gut [2].

The vulnerability of chickens to oxidative stress is a key problem in the current intensive poultry industry, and oxidative stress acts as a driver for pathogenesis in animals. Diseases as stressors have been reported to distort the redox balance of the gut [16]. Broiler production is often associated with the high incidence of diseases such as necrotic enteritis which are caused mainly by *C. perfringens*, *Salmonella*, *E coli*, and *Eimeria* spp., accounting for huge economic losses due to retreaded growth performance [17,18,19] and high mortality rate [20,21,22,23] accruing from the diseases. The severity of necrotic enteritis disease occurs mainly in the gut, causing intestinal epithelial damage and disrupted physiological functions of the gut; reduced immunity and high immune response [24,25]; decreased antioxidant capacity [14]; increased intestinal inflammation [26,27,28]; impaired intestinal barrier function [15,29]; alterations in villi morphology [13,27]; gut lesions [30,31,32]; and microbial dysbiosis [11,15,33], which ultimately impairs nutrient absorption and utilization. The control of gut-infection-induced oxidative stress and the utilization of gut enhancers to augment the resistance capacity of the host has become expedient. In the past, antibiotics have been utilized to promote gut health, but issues arising from bacterial resistance, detrimental environmental impacts and the safety of animal products in the food chain [34] renders it an obsolete therapeutic agent.

In a bid to promote the gut health of broilers for improved performance, natural microbial therapies such as probiotics have been proposed for utilization in the poultry industry as gut enhancers targeted towards the suppression of pathogens and the boosting of intestinal immunity [35]. Positive effects of probiotics on gut health from the perspectives of improved villi morphology [36,37,38], regulatory effects on immune and inflammatory response [39,40], the maintenance of intestinal epithelial integrity [41,42,43] and the modulation of intestinal microbiota [44,45,46] have been reported. In addition, probiotics can enhance intestinal development and integrity via the stimulation of intraepithelial lymphocytes and antioxidant capacity [47]. The encouraging results regarding probiotics are due to their multiple beneficial characteristics such as the competitive exclusion of the pathogenic bacteria [11,48], immunomodulatory capacity [49] and the production of volatile fatty acids and bacteriocins [50,51]. These findings provide evidence that probiotics could be used to prevent and control diseases in birds exposed to necrotic enteritis challenge with consequential effects on microbiota balance, antioxidant capacity, immune and inflammatory regulation and stabilized villi morphology. Probiotics have been demonstrated to mitigate the adverse effects of necrotic enteritis on gut health [12,13,52,53] and enhance recovery rates. The potency of probiotics in controlling and reducing the severity of necrotic effects is associated with the use of different probiotic strains, dosage supplementation and various mechanisms of action of probiotics. In this review, we therefore explored the adverse effects of necrotic enteritis on gut health from the view of infection rate and gut lesions, antioxidant capacity, immune and inflammatory response, shift in microecological balance, villi morphology and the regulation of intestinal barrier integrity with consequent effects on growth performance. Additionally, we provide an insight into the recent trends in probiotics strains that have been used to mitigate the effect of NE challenge and the underlying mechanisms of action.

## 2. Efficacy of Dietary Probiotics on Enhancement of Gut Health in Conventional Poultry Model

A stable gut structure with efficient physiological function is the key driver for nutrient absorption, utilization and fast translation to improved growth performance. Gut health consists of intact villi structures and intestinal epithelia, the absence of gut lesions and minimum pathogen load in the gut, redox balance between gut immune cells and inflammatory cytokines and balanced gut microbiota. Ample evidence exists in the literature on the significance of gut health in improved performance and reduced mortality rates in broiler production, which is of economic benefit to the poultry industry and boosts food security. The influence of dietary probiotics on various components of gut health are presented in Table 1.

### 2.1. Intestinal Villi Morphometrics 

Intestinal villi are unique finger-like protrusions on the inner wall of the small intestine, which have the function of absorbing nutrients. The zigzag pattern tissue structure of well-developed intestinal villi promotes nutrient absorption as it increases digesta retention time and allows more contact of nutrients with the absorptive surface of the intestinal epithelium [70,71]. Intestinal villi are covered structurally by the intestinal epithelium, with a continuous layer of myofibroblasts beneath the epithelium that maintains epithelial cells renewal and defensive mechanisms [72]. The differentiation and proliferation of the enterocytes occur at the crypt, and this enhances villi growth [73]; thus, a shallower crypt gives rise to longer villi, broader villi surface areas and more absorptive epithelial cells. Evidence exists that probiotics enhance the development of intact villi via enhanced enterocyte differentiation, increased villi height and villi height to crypt depth ratio and reduced crypt depth [28,39,40,58]; these structures facilitate nutrient absorption, which explains the improved growth performance.

### 2.2. Intestinal Epithelial Barrier

The optimal functionality of the gut is critical to animal health, welfare and performance, but this may be modulated by efficient gut barrier function [74]. An intact intestinal epithelium supports the GIT to function as a barrier between the host and the environment, thus preventing pathogen invasion and the translocation of molecules and antigens not beneficial to the host [10]. The intestinal barrier function is a network of regulatory pathways involving the vascular endothelium; the epithelial cell lining and the mucus layer; the immunological barrier, which consists of digestive secretions; antimicrobial peptides; cell products such as cytokines; inflammatory mediators and immune molecules synthesized primarily by Paneth cells in the crypts of the small intestine and trefoil factor family proteins [75]. The intact intestinal epithelial barrier is regulated by a host of molecules, pathways and enzymes including tight junction proteins, mucins, goblet cell numbers, enzymes (IAP and DAO) and oligosaccharides. TJs are the apical-most adhesive complexes that preserve the intracellular space and consist mainly of transmembrane proteins (e.g., claudins and occludin), peripheral membrane proteins (e.g., zonula occludens (ZO)-1 and ZO-2), and regulatory proteins. [76]. The tight junctions and adherens junctions are attached to the framework of actin and myosin which allows the regulatory mechanisms of the junctions to function via the cytoskeleton. Dietary probiotics have been reported to improve the integrity of the intestinal barrier and its functionality via the increased expression of tight junction proteins and enzymes, and the modification of tight junction structures, which culminate in reduced intestinal permeability, endotoxin translocation and inflammation [36,38,39,57]. The preservation of gut barrier integrity and the enhanced transcriptome profile of intestinal epithelial cells is a function of a stabilized microbiome and its metabolites [77]. The enhanced intestinal epithelium increases the resistance of the birds to diseases, improves gut function and sustains microbiota balance.

### 2.3. Immune Regulation

Intestinal immunity in the chickens is controlled by intraepithelial lymphocytes (IEL), which consists of natural killer cells, T cells, and B cells, which are components of gut-associated lymphoid tissue, secretory immunoglobulins, and host of other defensive mechanisms.

Secretory IgAs, or sIgAs, are antibodies produced by plasma cells residing in the intestinal lamina propria, and they represent the first line of defense against the entry of enteric toxins and pathogenic organisms [78]. Gastrointestinal sIgAs play a crucial role in the maintenance of the intestinal epithelial barrier and mucosal homeostasis, which in turn modulates the intestinal microbiota and affects the development of systemic immunity [79]. Also, immunoglobulins such as IgM, IgA and IgG are involved in regulation of intestinal immunity. IgM is involved in maintaining the intestinal epithelial barrier by reducing the level of endotoxin during inflammation [80]. IgG is an abundant subtype in serum and extracellular tissue fluid, accounting for 70–75% of total immunoglobulin, and it serves as an indicator for the systemic immune status of the animal and plays a role in increasing the growth rate and feed intake. IgA mediates several protective functions via interaction with specific receptors and immune mediators, thus preventing the binding of pathogens to the mucosal surface [2]. Intraepithelial lymphocytes (IELs) are made up of natural killer cells, T cells and B cells which are key components in gut-associated lymphoid tissue. B cells are well known for their role in antibody-mediated immune responses, their critical role in T cell activation via antigen presentation and cytokine production [81]. The CD4+ (helper) and CD8+ (cytotoxic) T lymphocytes subsets are components of cell-mediated immunity, and the stimulation of CD4+ and CD8+ T cells is vital and critical to the maintenance of cellular immune response in animals during exposure to stressors, including disease [49]. Cecal tonsils are major gut-associated lymphoid tissues in chickens and are responsible for inducing immune responses against pathogens. The immunomodulatory property of probiotics on the innate and humoral immunity of the host makes it an excellent immune regulator [35]. Dietary probiotics have been reported to enhance the secretion of serum immunoglobulins and intestinal IgA, and increase the population of T cells [36,37,40]; all these aspects boost the immunity of the host and increase resistance against infection and intestinal damage. However, significant effects of probiotics on immune regulation were not observed in some studies [57,59,64]; the observed variations may have been due to the strain used or the dosage of the supplement.

### 2.4. Regulation of Intestinal Inflammation

Cytokines play crucial roles in the modulation of inflammatory response in the gastrointestinal tract [82]. Proinflammatory cytokines such as TNF-α, IFN-γ, and IL-6 play a crucial role in the modulation of the inflammatory response caused by pathogens in the gastrointestinal tract. Proinflammatory cytokines can induce the endocytosis of tight junction proteins, resulting in increased intestinal permeability [30]. Anti-inflammatory cytokines such as IL-4, IL-10, and TGF-β have an important role in the control of the duration and magnitude of the inflammatory response by inhibiting the production of proinflammatory cytokines [82]. Proinflammatory cytokines such as IL-1β, IL-6, and TNF-α can induce the differentiation and proliferation of leukocytes to eliminate pathogens, and further to regulate immune response. TGFB1 is related to mucosal immune tolerance, and TNF-α is a key regulator of inflammation. IL-6 is an effective pro-inflammatory cytokine of Th1 cells, mainly secreted by intestinal epithelial cells. IL-1β is a strong pro-inflammatory cytokine that is secreted mostly by macrophages and is essential for innate responses to infections [83]. IL-12p35 mRNA is an inflammatory cytokine promoting Th1 responses and the production of IFN-γ [84]. IL-17 (a pro-inflammatory cytokine) expression increases in the intestine of chickens in response to infection with CP [85]; IL-17A is important in inflammation and antimicrobial defense against pathogens (extracellular bacteria and fungi) at mucosal surfaces and regulates mucosal immune defenses [86]. The potential of probiotics to reduce the production of pro-inflammatory cytokines and increase levels of anti-inflammatory cytokines has been reported [42,57,58]. The inflammatory regulatory function prevents paracellular permeability, the disruption of tight junction structures, increased immune response and consequent intestinal damage.

### 2.5. Modulation of Intestinal Microbial Composition

The bacterial species found in the chicken gut include four predominant bacterial phyla (*Proteobacteria*, *Bacteroidetes*, *Firmicutes*, and *Actinobacteria*) [87]. The gut microbiota is involved in the digestion and absorption of nutrients, contributing to the construction of the intestinal epithelial barrier, the development and function of the host immune system, and competing with pathogenic microbes to prevent their harmful propagation [88]. The microbial fermentation of carbohydrates results in the production of a range of Short chain fatty acids (SCFAs), predominately acetate, propionate, butyrate and lactate. The SCFAs, especially butyrate, are the preferred substrate for epithelial cells and are associated with cell proliferation, differentiation, and apoptosis; increased MUC2 gene expression; and antioxidant activity [89]. They all play a part in the integrity of the gut barrier. Proteins and complex carbohydrates are metabolized by the gut flora, which creates a wide range of metabolic products that can facilitate interactions between the gut epithelium and immune cells. Probiotics as natural gut enhancers have been found to cause a shift in the microecological balance of the gut; this shift often provides a conducive environment for the proliferation of beneficial microbes and the suppression of pathogenic bacteria [36,39,43,90]. A microbial composition consisting more of beneficial microbes and less of pathogens facilitates improvement in the physiological response of the host, ranging from immune response, nutrient absorption and metabolism.

Taken together, the findings imply that probiotics have the potentials to maintain gut health via the functional active intestinal epithelium. It is proposed that probiotics could mitigate the adverse effect of infection on gut health via increased gut integrity, intestinal villi development, the modulation of microbiota and immune and inflammatory responses. This provides the basis for the use of probiotics to ameliorate the severity of necrotic enteritis impact on gut health of broiler birds.

## 3. Necrotic Enteritis in Broiler Challenged Model

**Figure 1:**
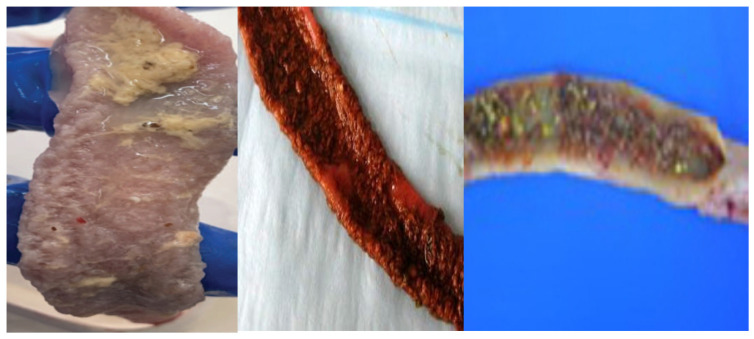
Damage of intestinal epithelia due to necrotic enteritis. Adapted from [93].

Necrotic enteritis (NE) is caused by mainly by *Clostridium perfringens* (CP) and typically occurs in broiler chickens between 2 to 6 weeks of age. NE pathogenesis is mainly attributed to the production of tissue-degrading toxins: NetB (major toxin), alpha (α)-toxin and TpeL by CP [91]. Clinical NE is characterized by high mortality in poultry, while subclinical NE (SNE), which is becoming more prevalent, is mainly characterized by intestinal mucosal damage without clinical signs or mortality [92]. Intestinal damage which reflects necrotic enteritis is presented in Figure 1.

Intestinal damage most often leads to the leakage of nutrients into the lumen of the small intestine, providing nutrient substrates for the rapid proliferation of *Clostridium perfringens* and causing damage to the intestinal tissues [94]. Intestinal NE lesions and mucosal atrophy greatly compromises epithelial permeability and mucosal barrier function, resulting in bacterial translocation to the liver, spleen and blood [33]. There exists a strong correlation between NE and poor feed conversion ratio and reduced growth performance in broilers [95]. NE has a serious impact globally on poultry production, causing severe economic losses due to reduced growth performance, increased mortality, huge treatment costs and poor flock uniformity. The incidence of necrotic enteritis is associated with the overgrowth of *Clostridium perfringens*, *Salmonella* spp., and even *Escherichia coli* in the GIT of poultry. For instance, it is thought that one of the key risk factors for NE outbreaks is coccidiosis, a parasitic disease of the chicken digestive tract brought on by coccidian protozoa of the *Eimeria* species.

*Clostridium perfringens* (CP) is a natural inhabitant of the poultry intestinal tract, which cohabits with other local microflora and causes no harm to the host. However, because of the higher growth rate of CP when compared to other intestinal microorganisms [96], its outgrowth could cause NE. In healthy birds, the population of *C. perfringens* is ~10^2^–10^4^ CFU/g digesta; however, disease occurrence significantly causes an increase to 10^7^–10^9^ CFU/g digesta [97]. The outgrowth of CP is associated with disruptions to the intestinal homeostasis and the production of toxins which cause cellular damage [98]. When pathogenic strains of CP are established at significant levels in the intestine, signs of necrosis in the intestinal epithelium, hemorrhage, diarrhea and consequently loss of performance may occur [99]. This is because *C. perfringens* enterotoxins (CPEs) bind to tight junction proteins, mainly claudin-3 and claudin-4, which increase mucosal surface pores, leading to an increase in paracellular permeability and cytotoxicity [100]. Mostly, coccidia, as a predisposing factor for CP, induces protein leakage, which supplies nutrients for the increased replication of CP and consequently damages the intestinal mucosa, leading to poor performance.

The *Salmonella* species is a pathogen that causes salmonellosis in humans and domestic animals. *Salmonella enterica* serotype Enteritidis (SE), a Gram-negative intracellular pathogen, is one of the most common serotypes of *Salmonella* bacteria reported worldwide and is the major source of human intestinal infections reported in recent years [101]. Salmonella, an enteric disease, easily colonizes the gut in newly hatched chicks and is found to be major cause of intestinal microbiota dysfunction and intestinal inflammation, which damage the intestinal epithelium, leading to poor performance and economic losses [102,103]. There are evidences that *Salmonella* infection retards growth performance and increases the colonization of salmonella in the host [27,104]. Challenge by pathogenic *Escherichia coli* is common in animal breeding and production, disrupts chickens’ intestinal tracts and retards growth performance [105]. *E. coli* is a Gram-negative bacterium, and its core pathogenic element is lipopolysaccharides, these endotoxins can trigger system inflammation and cause death. Inflammation limits the synthesis of muscle protein and mobilizes energy to support the immune response, resulting in poor growth [106]. Infection due to *Coccidia*, *Salmonella* or *Eimeria*, supports the invasion of CP into the mucosal membrane, leading to the onset of NE damage on the intestinal integrity.

### 3.1. Effect of Necrotic Enteritis Challenge on Gut Health of Broilers

**Figure 2:**
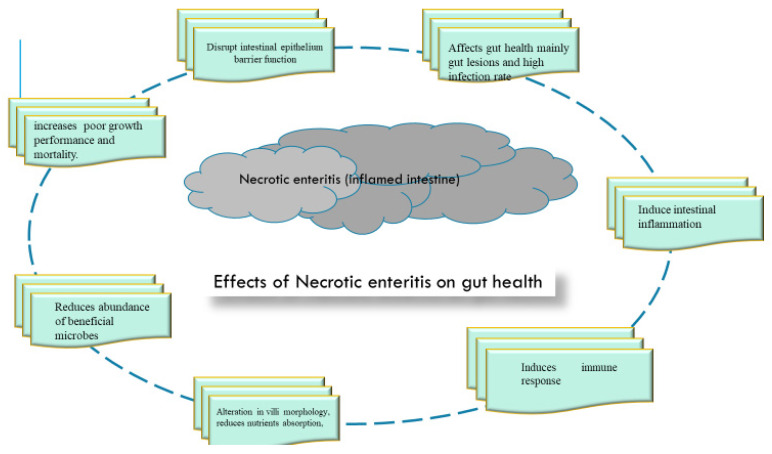
Diagrammatic representation of NE challenge effects on gut health.

The exposure of birds to infection disrupts the normal redox balance of the gut due to; disrupted intestinal integrity, lesions and pathogen loads in the gut, increased immune and inflammatory responses and altered microbiota in favor of pathogens. These obviously damage the intestinal mucosa, leading to retarded growth performance and high mortality rates. Providing an insight into the disruption of various gut health components due to disease challenge may offer a pragmatic direction on how to use these biomarkers in a non-invasive approach to monitor animal health. The effects of NE challenge on various components of gut health are presented in Figure 2.

### 3.2. Induced Gut Lesions and Pathogen Colonization

During exposure to disease challenge, the attachment of bacterial pathogens to intestinal epithelial cells causes the disruption of tight junctions, the rearrangement of the actin cytoskeleton and the alteration of intestinal microbial balance, leading to damage of the extracellular matrix and cellular junctions [107]. The resultant effect is a compromised intestinal epithelial barrier and increased paracellular permeability, which facilitates pathogen invasion, necrotic lesions and the translocation of bacteria and endotoxins to various organs such as the liver [108]. The induction of pathogen infection and lesions on various gut segments due to necrotic enteritis challenge is listed on Table 2.

In broilers exposed to NE challenge, higher levels of *C. perfringens* loads were notable in the ceca [25,26] and ileum [11,12,113,118,124]. The population of *C. perfringens* and *Escherichia coli* were found to be high in the ileum and cecum [125]. The CP invasion in the liver was higher in the NE-challenged flock compared to the normal flock [25,112]. Increased pathogen shedding increases the infection rate and horizontal transmission of pathogens between and within flocks. Pathogen invasion cause intestinal lesions and the translocation of endotoxins, which damage the intestinal mucosal layer. Lesion scores are commonly used as clinical indicators for the assessment of NE severity on the gut. The histological evaluation of NE-associated enteritis was significantly correlated with lesion score (gross pathology) [117], which validates the lesion scoring used in the field for the diagnosis of NE. Pathogen-induced intestinal lesions were notable in the small intestine [25,28,126], duodenum and ileum [20,23], jejunum [33] and ileum [110] of birds infected with *C. perfringens*. Additionally, footpad lesions associated with wet litter were notable in CP-challenged birds [113,124]. *E. maxima* (*Eimeria* spp.) caused severe lesion scores in the gut epithelium and increased oocyst shedding in the fecal samples [127]. The occurrence of higher lesion in the duodenum and jejunum compared to the ileum may be partly explained by the toxin-producing capacity of the pathogens. An in vitro study demonstrated that bile acids, which are secreted in the upper parts of the small intestine, cause higher secretion of *C. perfringens* type A enterotoxins [128]. The increased pathogen colonization and lesions in the gut lead to reduced growth performance and a high mortality rate, as listed in Table 2. The increased mortality rate in NE-challenged birds is mainly due to the accumulation of toxins produced by CP. Evidently, increased pathogen colonization and severe intestinal lesions would compromise intestinal epithelial barrier integrity, increase stressors in the gut, enable endotoxin translocation into the bloodstream and organs and alter the constituents of local and systemic lymphoid organs at the cellular level, thus impeding the nutrient absorption process and causing an increased mortality rate.

### 3.3. Disruption of Intestinal Epithelial Barrier Function/Integrity

The intestinal epithelium as a mechanical barrier is crucial for the absorption of nutrients, electrolytes and water, as well as the maintenance of intestinal barrier integrity, function and the protection of the gut from enteric pathogen invasion [76]. Tight junction proteins are the most important aspect of gut integrity and make up a barrier in the paracellular space. These proteins are subject to change and remodel in response to external stimuli in the gut lumen such as food/nutrients and commensal and pathogenic bacteria [129]. During NE challenge, the proliferation of *C. perfringens* spores and the increased production of *C. perfringens* enterotoxins (CPEs) results in signaling cascades that cause alterations in tight junction structures and damage the intestinal mucosa [10]. It has been previously shown that the CPEs recognize the extracellular domains of the claudin family proteins, mainly claudin-3 and claudin-4, in the tight junction structure as binding sites/receptors [130]. The attachment and increased paracellular permeability and cytotoxicity results in pore formation and the disruption of gut integrity in the host. Thus, the dysfunction of the epithelial barrier increases intestinal permeability, which is reflected in the increased or decreased expression of many biomarkers such as tight junction proteins, enzymes and intracellular proteins. The effects of NE on intestinal epithelial barrier function are listed in Table 3.

Previous studies have shown that during NE challenge, infection influences the expression of tight junction proteins either in increasing or decreasing trends. *C. perfringens* challenge reduced the OCLN mRNA expression but exerted no effect on the expression of CLDN1 or ZO-1 in the jejunum [125] and reduced the expression of claudin-1 but had no influence on occludin and claudin-2 [26]. The expression of occludin and claudin-1 was suppressed by *C. perfringens* and *E. coli*, respectively. [28,131] *E. coli* infection caused a downregulation of mRNA expressions of proteins (occludin and ZO-1) in the ileum [129]. Salmonella infection reduced the mRNA expression of *Muc2* and *Tff2* [15], mucins and claudins in both the jejunum and ileum ([132] and the expression of ZO-1 in the intestinal tissues [27]. Mucins, which are produced mainly by goblet cells, are often reduced in concentration due to a decrease in the number of goblet cells after infection [129]. The reduced expression of tight junction proteins and altered tight junction structure increases the paracellular permeability and consequent translocation of toxins. CRP, which is an acute-phase protein and a component of the innate immune system, is often considered as a metabolic inflammatory marker [133], while diamine oxidase (DAO), which is found in the small intestinal mucosa, is a marker for intestinal barrier function. Increases in serum concentrations of DAO and CRP [129] and endotoxin content [125] due to infection have been reported. The presence of these molecules in the bloodstream causes leaky gut and reduced gut function [28,134]. In addition, the increased mRNA expression of the intestinal MLCK gene (myosin light chain kinase) was reported in birds under NE challenge [112] and caused intestinal damage. This adverse effect is due to the fact that the intestinal MLCK gene (myosin light chain kinase) pathway is involved in both the degradation or distribution of TJs and intestinal permeability [135]. The activity of intestinal alkaline phosphatase (IAP) was reduced during exposure to disease [25]. The aforementioned findings depict the loss of intestinal barrier function, the impaired activity of brush border enzymes and villi development and consequent immune and inflammatory responses. The mTOR pathway may be involved in the protection of intestinal epithelia and the regulation of inflammatory response. In SNE-challenged birds, the downregulation of the mTOR pathway led to a reduction in the expression of tight junction proteins, which induced intestinal inflammation and intestinal epithelium damage [12]. The reduced expression of growth factors would impair intestinal cells’ renewal. In SNE-infected chicks, CP caused a significant reduction in the levels of IGF-1 in the jejunum and ileum and the level of EGF in the jejunum, which shows that poor intestinal development is a consequential effect [123]. Impaired intestinal development could be because, EGF, is mainly involved in enhancement of cell proliferation and restoration of damaged epithelium, and IGF-1 is crucial to activity of digestive enzymes, thus promoting intestinal cells growth.

It could be inferred that compromised intestinal barrier integrity due to NE challenge may be via the disruption of multiple intestinal TJPs, genes and adhesion molecules and the deactivation of enzymes and pathways involved in maintaining intestinal barrier integrity. Therefore, the consequential negative effect induces the alteration of villi morphological structure, intestinal inflammation, reduced immunity and altered microbial composition in the gut.

### 3.4. Alterations in Villi Morphology

NE challenge causes alterations in villi morphology, which is evidenced by severe hemorrhages in small-intestinal tissue, the proliferation of inflammatory cells, the shortening of the villus and an increase in crypt depth. These necrotic effects on the mucosal layer, goblet cells and enterocytes in the villi impair the absorption of available nutrients in the intestinal lumen, which in turn reduces performance and resistance to diseases. The effects of NE on the villi morphology of broilers are presented in Table 4.

Evidences from literature has shown that during NE challenge, CP infection causes damage to the intestinal villi structures, which is reflected in reduced villi length and increased crypt depth [13,25,70,117,125], and the decreased integrity of the lamina propria [70] and intestinal mucosal layer [13]. In the study of [118], the CP-induced NE challenge collapsed the mucosal layer of the small and large intestines, thus altering the structures of enterocytes and epithelial brush borders. In birds exposed to NE challenge, the ability of the CP to synthesize toxins such as NetB caused damage to the intestinal villi structures [113]. *E. coli* O78 disrupted the intestinal morphology of the infected birds and induced a high inflammatory response in the jejunum, leading to severe jejunal villi damage [131]. In *Salmonella*-infected birds, reduced jejunal goblet numbers [138], reduced villi length and shallow crypt in the jejunum and damaged intestinal mucosal surface [27,50,132,138] were notable. The damaged villi structure resulted in the reduced activity of digestive enzymes [50]. However, CP infection did not cause significant damage to the intestinal morphology [20]; it could have been that the dosage was for subclinical necrotic enteritis, not necrotic enteritis. *E. coli* had no influence on the villi height and VH/CD ratio, probably because the strain used did not disrupt intestinal morphology [129]. The effects of lesions, infection and impaired gut morphology are reduced weight gain and an increased feed conversion ratio.

### 3.5. Intestinal Inflammation

Proinflammatory cytokines are involved in the early response and amplification of the inflammatory response; the proinflammatory cytokines can activate the effector mechanism of the epithelium and maintain intestinal integrity [139]. During infection, these cytokines are overexpressed and rapidly released, disrupting the intestinal barrier function which would orchestrate chronic inflammation and cause intestinal permeability and severe damage to the intestinal mucosa. In birds exposed to NE challenge, CP-induced inflammation is via the activation of Th1, Th2 and Th17 cells and the inhibition of Treg cells, evidenced by the upregulation of IL-13 and IL-17 and the downregulation of TGF-β4. Anti-inflammatory cytokines (TGF-β, IL-4 and IL-10) and growth factors (EGF, GLP-2 and IGF-2) are downregulated during infection. The intestinal inflammatory response induced by these pathogens could be attributed to the TLR-4/NF-kB signaling pathway. The influences of NE challenge on the expression of proinflammatory and anti-inflammatory cytokines and pathways that regulate the production of cytokines are presented in Table 5.

Reduced expressions of TLR2, TLR4 and TNFSF15 culminated in increased intestinal inflammation [25], but no significant effect was reported in [26]. IFN-γ is produced by T helper cells and natural killer cells that stimulate macrophages to secrete oxidants with antimicrobial properties [140]. The increased expression of IFN-γ in birds exposed to CP infection caused intestinal damage [14,25,141]. CP increased TNF-α in the intestine [26,28], but no significant effect was reported in [26]. IL-6 and IL-1β are the key proinflammatory cytokines that regulate a host’s immunity against pathogens. Increased expression during NE challenge suggests induced inflammation. IL-17A is linked with the initiation of inflammatory diseases [85]. C. perfringens challenge increased the expression of IL-17A [26]. Inflammatory responses are activated through various pathways which are involved in the regulation of intestinal inflammation. The increased expression of TLR-4, NF-κB, IL-1β and IL-8 and so on is through the activation of the TLR4/NF-κB signaling pathway [142]. The pathways, TLR4-, MyD88-and NF-kB-, which are involved in intestinal inflammation were activated in SNE-challenged birds [109]. Wnt is involved in the regulation of intestinal stem cells, and b-catenin is at the downstream end of the Wnt pathway [130]; this pathway is involved in intestinal repair. In the study of [15], the suppression of this pathway due to infection is reflected in damage to intestinal villi and a reduced number of PCNA+ cells in the crypt. In addition, increased expression of Hypoxia-inducible factor-1 (HIF-1α) due to infection resulted in intestinal inflammation, because HIF-1α is known to suppress the activation of β-catenin, causing a downregulation of Wnt [103]. Taken together, these findings provide ample evidence that NE challenges induce inflammatory responses via the activation of inflammation pathways, inflammatory genes and proinflammatory cytokines and an increased number of immune cell populations (heterophils, lymphocytes, macrophages and plasma cells), which invariably lead to damage to epithelial barrier integrity and the intestinal submucosa.

### 3.6. Intestinal Immunity

Host–pathogen interactions during NE are complex and involve different components of the host immune system [143]. In the face of infection, an immune barrier to pathogen invasion is provided by the gut-associated lymphoid tissue, which is made up of different cells that release pro- and anti-inflammatory cues to maintain gut homeostasis [78]. Infection disrupts the immune system of the host due to the stimulation of various cells which act in response to the oxidative stress induced by pathogen infection. The influences of NE challenge on intestinal immunity are listed on Table 5.

The exposure of birds to NE challenge caused variations in the number of T cells [24,26,129]. The concentrations of various immunoglobulins were reduced by pathogens during NE challenge [14,24]. Secretory IgA is a major component of the intestinal mucosal barrier and plays an integral role in intestinal protection [144]. NE challenge caused a reduction in the level of sIgA [25,95,110]; such a reduction enhanced pathogens’ adhesion to sites on the mucosal surface. An increase in IgA due to a challenge would be due to intestinal damage, which stimulates local inflammatory responses, which then increases the production of inflammatory responses [145]. The increased expression of TRAF3, an immune signaling molecule, could promote an inflammatory response against the co-infection of *Eimeria* and *C. perfringens* during NE challenge. The reduction in immunity may be enhanced via mRNA expression levels of MMP-2; MMP-2 is known to be involved in the collagen degradation of soft tissue, thereby destroying the lamina propria and in turn reducing immunity due to decreased lymphocytes [14]. CP infection reduced mRNA levels of mucin-2, LYZ and fowlicidin-2 [25], leading to reduced intestinal immunity. The activation of immunity response due to infection is an energy-consuming process which diverts energy needed for growth to the development and activation of immune cells, thus retarding growth performance.

### 3.7. Intestinal Microbiota

The richness of gut microbial diversity is an indicator for good health, while decreased richness acts as a predisposing factor for intestinal dysbiosis and other complications. Enteric infections are known to cause an imbalance in the resident commensal population while promoting gut colonization by the pathogenic bacteria. The decreased diversity of gut microbial species enhances pathogen colonization in the gut and increases the susceptibility of the host to diseases due to reduced resistance [146]. A significant decline in microbial diversity was prevalent in birds under NE challenge [19,32,33], and CP caused bacterial dysbiosis in the cecal contents [25]. This reduction in microbial diversity depicts intestinal microbial dysbiosis due to infection. The effects of NE challenge on gut microbiota are listed in Table 6.

It has been reported that *Firmicutes*, *Cyanobacteria*, *Proteobacteria*, *Bacteroidetes* [14], *Firmicutes*, *Proteobacteria*, *Bacteroidetes*, *Tenericutes* and *Verrucomicrobia* [147] are the major bacterial phyla in the ileal and cecal microbiota, respectively. The *Proteobacteria* phylum consists of various pathogens, including *Shigella*, *Salmonella* and *Escherichia coli*, which are relatively abundant in hosts exposed to infection. These pathogens are linked with intestinal damage as they colonize the gut; this could partly explain the significant mucosal damage in birds under NE challenge. Various studies demonstrated that infection increased the abundance of *Proteobacteria* [132,148], *Bacteroidetes* [15], *Lachnospiraceae* and *Enterobacteriaceae* [132], and there were higher levels of ileal *Bacteroidetes* and cecal *Proteobacteria* [33] in the birds. The abundance of *Prevotellacea*, *Clostridium*, *sensu stricto* 1. and *Muribaculacea* in birds with SNE has been reported [23]. *Bacteroides* and *Prevotellaceae* can degrade mucus oligosaccharides, resulting in the disruption of the intestinal mucosal barrier and intestinal inflammation [149], which coincides with increased gut lesions and reduced performance. During infection, there is always a shift in birds’ microbiota, which is reflected in increased *Bacteroidetes* and a reduction in *Firmicutes*, although this is sometimes inconclusive. Gram-positive *Firmicutes* phyla harbor many health-promoting bacterial groups such as *Lactobacillus* and are recognized as a primary pool of probiotic species [79], and *Firmicutes* and *Bacteroidetes* are linked with butyrate production [150]. A reduction in the relative abundance of *Firmicutes* due to the negative effect of NE challenge suggests the proliferation of pathogens and is linked with intestinal damage. The decreased abundance of *Lactobacillus* species, *Ligilactobacillus*, *Lactobacillus* [15], *Lactobacillus salivarius* [26] and *Lactobacillus* [53], in infected birds is not beneficial to the gut health of the host. However, CP infection reduced *Bifidobacteria* populations in the ileum but had no effect on the composition of *Lactobacillus* [126]. In another study, the populations of *Ruminococcus* spp. and *Bacillus* spp. were significantly reduced [11]. The depletion of butyrate-producing bacteria in the gut results in increasing inflammatory damage; this is because these bacteria have anti-inflammatory and epithelial-barrier-strengthening effects. In SNE-challenged chickens, the reduced abundance of *C. cluster XIVa* in the jejunum and ileum and *C. cluster IV* in the ileum was notable [123]. The decrease in phyla of beneficial microbes increases pathogens’ adhesion to the intestinal walls and the susceptibility of the host to infection and intestinal damage. Intestinal damage in NE-challenged birds is credited to the fact that CP, the main pathogen, produces toxins that accumulate in the GIT and cause intestinal permeability, allowing endotoxins to enter the bloodstream and harm chickens. Therefore, microbial shift in favor of pathogen proliferation facilitates intestinal damage, which coincides with compromised intestinal integrity, increased lesion, reduced gut fermentation and the synthesis of short-chain fatty acids. Consequently, reducing growth performance due to impaired nutrient absorption arising from impaired gut health.

### 3.8. Reduced Antioxidant Capacity, Metabolites and Nutrient Transporters 

The GIT is the main source of reactive oxygen species (ROS); the imbalance between oxidant and antioxidant systems leads to the excessive production and accumulation of ROS, which causes oxidative damage to biological membranes [151]. Oxidative imbalance acts as a key driver for inflammation [114]; thus, oxidative stress may play a key role in the pathogenesis of NE. SNE infection reduced the antioxidant capacity of the small intestine via the reduced activity of antioxidant enzymes and an increased MDA level, and it also activated the caspase-dependent apoptotic pathway, which upregulated the expression of apoptotic-related proteins and the occurrence of intestinal apoptosis [14,70]. CP increased the MDA level and suppressed the activities of SOD and CAT [30]. A reduction in the antioxidant capacity of the host increases the susceptibility of the host to infection due to damage to the biological membrane. *Salmonella* infection reduced the levels of total volatile fatty acids, acetic acid and butyric acid [148]; the reduced concentrations favored pathogen invasion. In SNE-challenged birds, lower levels of lactic, succinic, α-hydroxyisobutyric and malic acid and increased levels of indole and monoethanolamine were observed in the cecal contents [95], which made them susceptible to pathogen invasion.

All in all, necrotic induced-disturbance to gut homeostasis causes; intestinal lesions which favors pathogen invasion and damage of intestinal epithelium. Various mechanisms may be involved including; reduction in the expression of tight junction protein and alteration of tight junction structure, bacterial and endotoxin translocation, increased level of proinflammatory cytokines, microbiota shift in favor of pathogen proliferation, impaired villi development and reduced immune status.

## 4. Ameliorative Effect of Probiotics Supplementation on Necrotic-Enteritis-Induced Oxidative Stress in the Gut and the Underlying Mechanism of Action

**Figure 3:**
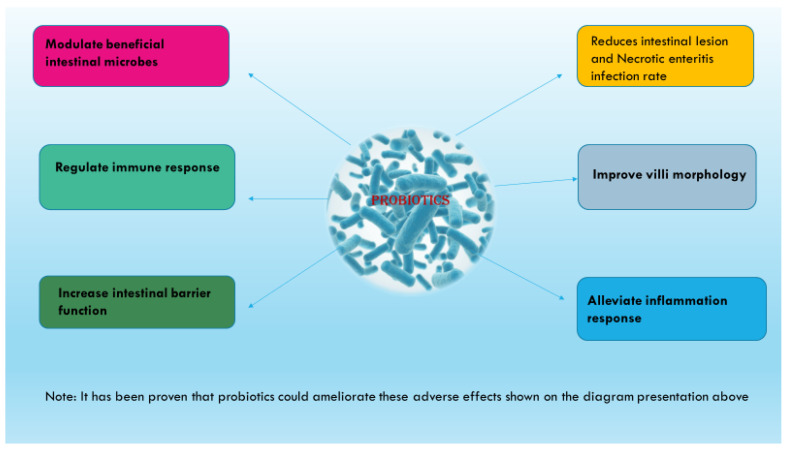
Mechanisms by which probiotics exert mitigative effects on gut health of birds exposed to NE challenge.

The utilization of microbial-based therapy potentiates ameliorating effects on NE-challenged broilers with better economic benefits [152]. The use of probiotics aims to improve intestinal integrity and overall gut health in disease-challenged broiler birds and increase the recovery rate for enhanced growth performance and reductions in economic losses. The probiotics’ mitigative effect could be through various mechanisms: reductions in gut colonization by pathogens and gut lesion, the regulation of intestinal inflammation, the protective effect on intestinal epithelial barrier integrity and villi structure, improved immunity and the alteration of gut microbial composition in favor of beneficial microbes. These positive effects hinge on antimicrobial and toxin detoxifying effects due to the synthesis of SCFAs and bacteriocins, the suppression of proinflammatory cytokines and the increased production of anti-inflammatory factors, the modulation of tight junction proteins’ expression and structure and villi development via the suppression of the apoptosis of intestinal epithelial cells. Various mechanisms by which probiotics promote gut health in NE-challenged birds are presented in Figure 3.

### 4.1. Pathogen Exclusion and Reduction in Intestinal Lesion Scores

The supplementation of probiotics in broiler diets has been proven to establish a balanced intestinal microbiome and increase the disease resistance of the host due to probiotics’ capacity to aid the preferential colonization of the gut via beneficial microbes while inhibiting the proliferation of opportunistic pathogens. The ameliorative effects of various probiotics on gut pathogen proliferation and lesion scores are presented in Table 2.

In birds exposed to NE challenge, the bacterial load of CP, which is the main causative pathogen, was significantly reduced in the gut via supplementation with probiotics strains; *Bacillus* [12,32,113], *Clostridium butyricum* (CB) [26] and bacteriophage [118]. The reduced pathogen proliferation is credited to the inhibition of attachment sites on the intestinal mucosal site via competitive exclusion by probiotics and the lytic activity of the phages. The pathogen exclusion or inhibition of CP proliferation could explain the reduced lesion score in the gut and mortality rate and improved the feed conversion ratio in birds under NE challenge but fed probiotics. Necrotic enteritis is characterized by gut necrotic lesions, which damages the intestinal mucosa and causes inflammatory cues that impair gut function. The positive effect of probiotics on the reduction of gut lesions in birds exposed to NE challenge have been well-documented in various studies [20,23,32,153]. In the same vein, bacteriophage [118] and antimicrobial products such as surfactin (obtained from the fermentation of *B. subtilis* and *B. licheniformis*) have exerted same effect [119,120]. Probiotics reduced the translocation of bacteria to the liver [21,112], and Bacillus *amyloliquefaciens* CECT 5940 [124] and *B. subtilis* DSM 32315 [113] reduced footpad lesions in infected animals, probably due to probiotics’ effects on dry litter quality. However, *Clostridium butyricum* [26], *Bacillus* spp., [17] and *B. subtilis* C-3012 [141] had no reduction effect on gut lesions. Also, a blend of feed additives had no effect on lesion scores [91]; probably, the level of infection can partly explain the variation. The decreased gut lesions due to dietary probiotics may be related to an array of probiotic benefits, including the production of beneficial antimicrobial compounds, the immunoregulation of inflammation and the stimulation of intestinal microbiota homeostasis, leading to enhanced intestinal health status. The literature has shown that positive effects on reduced pathogen colonization and lesion score could be due to anti-clostridial factors synthesized by *B*. *subtilis* PB6 [154], the enhanced expression of CLDN-3 which enhances gut integrity [53], the inhibition of pro-inflammatory cytokines’ production and the increased production of anti-inflammatory cytokines [25] and the reduction in the population of the alpha toxin producing *C. perfringens* in the ileum, which is the main site for NE challenge [113]. Probiotic strains are associated with the synthesis of bacteriocins and lactic acid [50] and the synthesis of SCFAs via *Bacillus* spp. (BS21 and BL26) [20], which creates an unfavorable environment for pathogen colonization. The study of [51] revealed that *B. licheniformis*-fermented-product-derived antibacterial cyclic lipopeptide surfactin can disrupt the bacterial membrane. This antimicrobial activity could lead to the death of *C. perfringens* and suppress the growth of *C. perfringens* in vitro. The antimicrobial effect of probiotics entails strong adherence to epithelial cells of the gut, which prevents opportunistic pathogen invasion, the persistence of the probiotic spp in the gut and the production of biofilms, which exert protective effects on the probiotics against gastric juices. The exogenous enzymes produced by probiotic strains could reduce the availability of nutrients for the nourishment of CP and proliferation, thus improving the microbial environment. All of these effects result in reduced lesion scores and oocyte shedding. Pathogen exclusion and reductions in gut lesions provide support for the intact intestinal epithelium, which culminates in proper nutrient absorption and utilization, leading to better growth performance and a faster recovery rate.

### 4.2. Improvement in Villi Morphological Structure 

Improvement in villi morphometry suggests an increased intestinal absorption area and the number of mature intestinal epithelial cells which are less susceptible to pathogen invasion and the translocation of endotoxins. Improved intestinal morphology may be a key factor to resistance against NE and consequently reduce the mortality rate, lesion score and growth performance (weight gain and reduced FCR). The ameliorative effects of various probiotics on villi morphology are presented in Table 3.

Dietary probiotics have been demonstrated to restore villi morphology when it collapses due to necrotic lesions. A reduction in pathogen proliferation would significantly decrease gut lesions and alterations in gut morphology. Surfactin, an antimicrobial peptide derived from probiotics’ fermentation, was found to enhance villi morphology due to its positive effect on the amelioration of gut lesions [119,120]. Bacteriophage restored the villi morphology and mucosal surface of birds under CP infection [118]; the ameliorative effect is due to antimicrobial activity against CP pathogens, leading to decreased toxin production, including those encoded by netB. Probiotic strains, including *Bacillus* [32,95,109] and *L. acidophilus* [13,125,129], restored the gut morphologies of NE-challenged birds. Contrarily, *B. subtilis* C-3012 did not enhance the ileal morphology in CP-challenged birds [141]. The intact gut villi reduce the rate of epithelial cell proliferation and tissue turnover in the crypt region, which would enhance the villi length and crypt depth, and the increased V/C ratio translates into a longer villus with matured epithelium cells for improved functions. Improved villi morphology allows for optimal intestinal barrier function and absorptive capacity, which are key drivers for increased growth performance and immunity. The above-mentioned evidence indicates that better intestinal development might be related to the preventive effect on SNE. Probiotics enhance normal villi architecture and protect the villi from enterotoxigenic CP infection by decreasing irritation and preserving barrier integrity. Improved villi development after challenge implies that probiotics can reduce the rate of epithelial cell proliferation and tissue turnover in the crypt region. The reduction in the enteritis index via probiotics, as measured using histopathology [117], suggests that probiotics can mitigate inflammatory effects. The improved microvilli architecture due to probiotics may be due to their effect on enterocyte surface architecture. Probiotics can enhance the gene expression of cytoskeleton proteins which maintain cell function and integrity. Improved intestinal dimensions may facilitate the capacity of intestinal walls to secrete a number of different compounds, including MUC2 and MUC3, which prevent the growth of harmful bacteria [155]. The ameliorating effects protect the intestinal epithelium and enhance the integrity of the intestinal mucosa. The development of an intestinal structure due to probiotics’ effects may be because probiotics can promote microflora; thus, these microbes may be involved in the activation of cell mitosis [156]. The capacity of probiotics to ameliorate the adverse effects of infection on villi morphology is achieved through various mechanisms, which include pathogen exclusion, the increased secretion of mucins and other biomolecules needed for intestinal cells renewal and proliferation and the suppression of inflammatory responses.

### 4.3. Regulation of Intestinal Epithelial Barrier Function/Integrity

Intestinal barrier integrity is crucial to the normal physiological function of the gut and overall gut health. The application of probiotics tends to exert a protective effect on the gut against pathogens and the permeability of toxins and oxidants in disease-challenged birds. The resulting intact intestinal epithelium results in normal nutrient absorption and utilization, which maintains the health of animal bodies. The barrier function is affected by various luminal and systemic cues that result in intestinal permeability, which promotes the translocation of plasma proteins and endotoxins. This enhanced intestinal barrier function could be achieved through various ways, including the upregulation of tight junction proteins, related genes and adhesion molecules and the activation of pathways that enhance intestinal barrier integrity for efficient nutrient absorption.

BS15 supplementation enhanced the contents of IGF-1 and EGF both in the jejunum and ileum of SNE-infected chicks, suggesting an ameliorating effect from intestinal injury [123]. *B. coagulans* increased jejunal goblet numbers but had no influence on the mRNA expression of Muc2 [25]. The capacity to increase goblet cells in challenged birds shows that probiotics could exert protective effects on the intestinal barrier against pathogen invasion and adhesion, thus reducing stress and improving gut health. The increased expression of JAM2 [122] and Fowlicidin- 2 gene mRNA levels [25] suggests the improved intestinal immunity and integrity of TJs.

Probiotics may alleviate intestinal damage due to NE by the modification of tight junction protein structures and increases in the expression of TJ proteins. Probiotics (Propal: Multi strain) enhanced the mRNA expressions of claudin-3 and zonula occluden-2 in the jejunum of NE-challenged birds [94], and the increased expression of CLDN-3 would reduce intestinal injury and improve mucosal integrity. *L. acidophilus* supplementation enhanced the mRNA expression of occludin, ZO-1 and claudin in the jejunum and occludin and ZO-1 in the ileum of *E. coli*-infected birds [129]. A probiotics complex attenuated intestinal mucosal barrier damage due to *S. typhimurium* challenge via the upregulation of tight junction proteins and goblet cells, and downregulation of the mRNA expression of Muc2 and Tff2 [15]. *Clostridium butyricum* ameliorated the production of *Muc2* which was disrupted due to salmonella challenge and increased the expression of tight junction proteins (ZO-1) and IECs [27]. CB was found to elevate the expression of claudin-1 in C. perfringens-challenged chickens [26]. *B. licheniformis* (BL26) increased the mRNA expression of claudin-1 in the duodenum and jejunum and ZO-1 in the ileum compared to *B. subtilis*, which suggests strain differences [20]. Occludin and ZO-1 are linked to the rejuvenation of the intestinal barrier and the stability of gut barrier function [76]. ZO-1 is a key protein linked to intestinal epithelial health and serves as a measure of the intestinal mechanical barrier [157]. Therefore, the increased expression of these proteins is critical for an intact intestinal epithelium and reduced paracellular permeability. However, *Lactobacillus* did not influence the expression of CLDN1, OCLN or ZO-1, and a decrease in MUC2 mRNA expression was notable [125]; probably, the strain used in the study did not benefit the host by enhancing tight junctions. The study of [110] reported that probiotics had no effect on CLDN-1. The positive effect of probiotics on the intestinal epithelium of birds under NE challenge could be via reductions in DAO and D-Lac contents [158] and endotoxins [141], which are linked to protective effects on the intestinal epithelium against CP-induced damage. The reduced levels of endotoxins due to NE by CB indicate that probiotics maintained intestinal function. The reduction in markers of intestinal permeability due to probiotics would promote intestinal integrity and reduce intestinal permeability, thus enabling stronger intestinal barrier function against SNE [159]. Ballooning is one of the main consequences of gut dysbiosis, and characteristics include the significant enlargement of gut diameter and an abundance of liquid, slime or gases, and it is often induced by the presence of gut pathogens [160]. In CP-challenged birds, *B. amyloliquefaciens* CECT 5940 notably reduced the abnormal content, ballooning and inflammation [124], probably because of the reduced CP population in the gut.

Mucins are binding sites for most pathogens, as they serve as sources of nutrients for their proliferation and as such induce changes in mucin expression. Improved MUC-2 expression would increase the protective effect of intestinal mucosa against pathogens and endotoxins, which would explain low levels of DAO [159]. *Lactobacillus* spores such as *L. johnsonii* and *L. reuteri* induced the expression of heat shock proteins (HSPs) and tight junction proteins, which limits bacterial adherence to the intestinal wall [161]. HSPs exert intestinal homeostasis and repairing effects in cases of bacterial infection [162] and play a role in the expression of IL-10 by intestinal epithelial cells, which in turn act as anti-inflammatory cytokines [163]. In one study, increasing Muc2 gene expression in mice with colitis by adding butyrate and acetate to drinking water improved gut chemical barrier function [164]. An increase in mucin production due to dietary probiotics may be linked with the synthesis of SCFAs, butyric acid can enhance the mRNA expression of mucins suppressed by NE, and increased butyric acid may be a mechanism by which it can increase mucin expression. However, intestinal mucus serves as a nutrient for CP which increases its proliferation and attachment to mucosal surfaces. Probiotics reduced the expression of Muc-2 [95]; these may reduce the mucosal colonization of CP and thus exert an protective effect against NE. Intestinal integrity is maintained when intestinal epithelial cells can be rapidly renewed after infection; *B. licheniformis* H2 upregulated the expression level of mTOR, which would accelerate intestinal epithelial cell renewal after infection [12]. The mechanistic target of the rapamycin (mTOR) signaling pathway is a key factor that regulates the renewal of intestinal epithelial cells along the crypt–villus axis [165]. It is probably achieved by exerting antioxidant effects and enabling the protein synthesis in the intestinal epithelial cells. 

Conclusively, selective infiltration of nutrients, endotoxins, pathogens into the intestine are orchestrated by intestinal barrier mucosa, thus protecting the intestinal integrity. Therefore, expression of various regulatory molecules that maintain intestinal barrier is a key to the ameliorative effect on the gut during NE challenge.

### 4.4. Regulation of Intestinal Immunity

The imbalance of the immune system is the main cause of excessive inflammation in infectious diseases; so, maintaining innate and systemic immune balance, which can be achieved with nutritional interventions such as probiotics, may aid to obtaining satisfactory results. The capacity of probiotics to exert immunomodulatory effects would favor the host performance, as nutrients are directed towards growth rather than stimulating immune responses.

Intestinal IgA production provides essential mucosal immunity against microbes as well as the suppression of inflammatory processes and the augmentation of general defense mechanisms. High IgA promotes intestinal repair, and a reduction in IgA due to probiotics may reduce inflammatory response, exerting an anti-inflammatory effect on the intestinal epithelium. Probiotics enhanced the sIgA levels in birds exposed to CP infection [25,95,159]. The abundance of sIgA in the probiotics group suggests that probiotics can lower the immune response and protect intestinal mucosa against the invasion of CP. In one study, probiotics increased the number of BU1 + IgA +, BU1 + IgM + and BU1 + IgY + cells in the spleens of challenged birds [13], which suggests that cellular immunity can be enhanced by the proliferation of B cells and lymphocytes. *B. subtilis* [126] and *E. faecium* [26] did not exert any effect on the SIgA level in the ileal mucosa of birds challenged with CP and *E. coli*, respectively. *Lactobacillus johnsonii* enhanced (IgG and IgA levels in the ileum), and the antioxidant capacity in the ileum which triggered anti-inflammatory cytokine production [114], and *L. johnsonii* (BS15) increased IgA+B cells in the lamina propria and levels of IgA, IgG and sIgA in the ileum [14] during CP-induced SNE challenge, thus increasing the immunity status of the challenged birds. CB had no influence on the intestinal IgA of CP-challenged birds [26]; this may have been due to the dosage level. Probiotics enhanced the population of CD3 + CD8α + T cells in the cecal tonsil [13] and CD3^+^ T cells in the intestine [33,125]. This induced an intestinal immune response via T cells against CP infection in NE-challenged birds. *E. faecium* had no effect on the number of CD3+, CD4+, CD8+, and CD4+/CD8+ cells on T lymphocytes (ConA S1) but increased B lymphocyte proliferation, as indicated by LPS SI. in the *E. coli* O78-infected birds, [131]; this implies that probiotics may enhance immunity response via humoral immunity rather than cellular immunity. Probiotics increased TLR2 and TLR4 expression [13]; the increase in TLR4 may have been linked to the increase in the Gram-negative *Bacteriodes*. The enhanced intestinal mucosa immunity effect of BS15 on birds with SNE challenge could also have been due to the downregulation of the mRNA expression of MMP-2, hence protecting the lamina propria [70].

Immunity response due to CP infection is an energy-consuming process, because it requires the synthesis of many new molecules and undertakes numerous cellular tasks, and it must occur rapidly [166]. Therefore, the immune system diverts nutrients from growth to ensure sufficient energy for an effective response (resistance), subsequently reducing growth performance. The expression of PGC-1α depicts an increase in cell energy metabolism. *B. licheniformis* H2 effectively reduced the expression of PGC-1α in challenged birds [12]; this reduction in energy metabolism would strike a balance between maintaining immunity and sustaining growth performance. The immunomodulatory effect of probiotics in animals challenged with diseases enhanced the recovery rate and suppressed inflammatory cytokines via the upregulation of T cells and immunoglobulins. Improved immunity status, which entails a balance in both cellular and humoral immunity, would reduce the production of inflammatory cytokines, protect the intestinal epithelium and promote nutrient utilization for better growth performance.

### 4.5. Regulation of Intestinal Inflammation 

Probiotics have been shown to help create an anti-inflammatory environment in the gut and reduce the production of proinflammatory cytokines. Probiotics not only improve intestinal innate immune-defense response against infection via the modulation of the TLR signaling pathway but sustain an intestinal immune balance which would prevent excessive inflammation through the regulation of anti and pro-inflammatory cytokines.

The capacity of probiotics to reduce the expression of IFN-γ and IL-6 [159] and increase the expression of IL-17 [94] would reduce systemic inflammation and intestinal damage. IL-17 is involved in epithelial cell regeneration and increased expression reduces gut lesions, thus exerting a protective effect on the mucosal surface. Probiotics’ downregulation of IL-12p35 mRNA transcript levels and IL-17 and IL-1β levels and increases in IL-13 and IL-2 [13] are anti-inflammatory mechanisms used by probiotics to protect the intestinal mucosa against NE infection. There exist evidences that probiotics upregulate the expression of IL-10 [94,159], which partly explains the mitigative effect of probiotics on intestinal damage. This is probably because during infection with protozoa and bacteria, IL-10 acts as an immune regulator and ameliorates excessive Th1 and CD8+ T cell responses, critical to the restoration of the epithelial barrier [167]. In addition, IL-10 is an important inflammatory cytokine, and its downregulation means that probiotics can mitigate inflammatory responses [159]. The inhibition of IL-1β secretion would lead to the enhanced expression of TJs, thus preserving the intestinal barrier integrity to a certain degree. The downregulation of IFN-γ, IL- 10 and IL-17 mRNA abundance occurred in the jejunum of broilers with subclinical NE, indicating an inhibition of Th2, Th17 and Treg cell function [53]. In the study of [33], probiotics exhibited stimulatory and inhibitory effects on cytokines production, which enhanced the recovery of birds from intestinal damage after NE challenge. CP beta toxins can stimulate *TNF-α* and *IL-1β* levels [168]. *L. plantarum* 1.2567 inhibited the mRNA expression of *chTNF-α* and *IL-1β* cytokines [30]. Probiotics’ inhibitory effect on these biomolecules may be related to their capacity to suppress neutrophil release and reduce inflammatory mediators [30]. Dietary probiotics increased the gene expression of *IL-10* and *TGF-β4* cytokines, suggesting that probiotics upregulate anti-inflammatory functions against *Salmonella* infection [132]. Feed supplements such as probiotics have been used to reduce intestinal inflammation and pro-inflammatory cytokines produced by CP infection through the inhibition of the TLR-4/ NF-kB signaling pathway. *B. licheniformis* activated the TLR-NF-kB signaling pathway but had no influence on IL-1b, IL-10, IL-17 or TNF-a [19]. This may have been due to the physiological functions of various probiotic strains. Altogether, the regulatory effect of probiotics on intestinal inflammation may involve striking a balance between the upregulation of anti-inflammatory cytokines and the downregulation of proinflammatory cytokines. In addition, the increased expression of Matrix metalloproteinase-2 (MMP-2) and nitric oxide (NO) due to CP infection is an indicator of severe intestinal damage due to inflammation; the reversal effect due to dietary probiotics ameliorated the adverse effect and preserved intestinal epithelium integrity [14,30]. This is because reduced expression of MMP-2 would decrease the collagen degradation of soft tissues [169], while reduced NO would decrease the number of mononuclear cells at the site of inflammation, which all contribute to intestinal damage.

The elimination of pathogen-induced gut inflammation is a key target for enhancing gut health, and probiotics, as natural antioxidants, may exert protective effects on the intestinal mucosa against inflammation. Previous literatures have demonstrated the capacity of probiotics to inhibit the proliferation of CP and support intestinal microflora balance, that leads to reduced inflammation [113,117,153]. Thus, the positive regulatory effect on intestinal inflammation may be attributable to intestinal microbiota modulation. This improvement in intestinal architecture would invariably reduce intestinal permeability and provide strong intestinal barrier integrity.

### 4.6. Antioxidant Capacity

Probiotics possess the potential to scavenge ROS, thus reducing the accumulation of ROS, which causes tissue damage and reduced resistance to disease [170]. *B. licheniformis* H2 increased the activity of antioxidative enzymes (serum: SOD, GSH, CAT and T-AOC; ileum: SOD, CAT and T-AOC) [109], and effectively suppressed apoptosis by increasing the Bcl-2 family proteins. PGC-1α regulates oxidative metabolism through increasing mitochondrial function and reduces the accumulation of reactive oxygen species [171]. The increased mRNA expression of PGC-1α and mTOR in the probiotics group [109] suggested higher capacity for metabolic regulation, which supports better growth performance. Probiotics enhanced IHR, T-AOC, CAT and SOD in animals under SNE challenge [24]; the enhanced antioxidant capacity would prevent oxidative damage to tissues. The mRNA expression levels of Nrf-2 were enhanced by probiotics, which suggests improved antioxidant function [14]. mTOR regulates protein and lipid synthesis; thus, its signaling is of significant/central importance in regulating cell metabolism, growth, proliferation and survival [172]. The renewal of intestinal epithelial cells along the crypt–villus axis is regulated by the mTOR signaling pathway. This may partly be due to its ability to affect the antioxidant capacity and protein synthesis of intestinal epithelial cells [76]. *L. plantarum* 1.2567 treatment enhanced SOD and CAT activities and decreased the MDA contents, proving that probiotics can enhance the antioxidant defense system by promoting the activities of antioxidant enzymes [30].

### 4.7. Modulation of Intestinal Microbiota 

The intestinal microbiota plays a key role in the host’s overall health, because these microbes are involved in regulating the proliferation of beneficial and pathogenic microbes, epithelial barrier function immune cells, pro- and anti-inflammatory cytokines, nutrient metabolism and pathways. In turn, these exert effects on growth performance. Probiotics and their metabolites enhance the symbiotic balance of microorganisms in the gut, thus providing a microbial community that is critical to animal nutrition and health [173]. Gut microbiota modulation by probiotics suppresses the population of pathogenic bacteria, which are inflammatory and immune inducers, while increasing beneficial microbes which are linked to the regulation of immune and inflammatory responses, exerting a wide range of health benefits on the host. This modulatory effect is one of the mechanisms by which dietary probiotics alleviate the adverse effect of NE challenge in birds and promote their recovery.

There is evidence that probiotics are key regulators of intestinal microbiota composition. *Weissella thailandensis*, a class of lactic acid bacteria (LAB), is most commonly used as a probiotic and plays key roles in disease resistance [174]. *Pediococcus acidilactici* can act as a potential probiotic as it produces lactic acid and bacteriocins against other enteric pathogens [175]. CB increased these spores [26], which shows the beneficial effect on the microbiota of NE-challenged birds. Similarly, members of the *Bacterioides* genus, including *Bacteroides thetaiotaomicron*, are involved in carbohydrate metabolism and the maintenance of desmosomes at the epithelial villus, promoting the GI tract’s integrity [176], hence, increased relative abundance is beneficial to the host. The relative abundances of *C. cluster IV* and *C. cluster XIVa*, which was previously suppressed due to SNE challenge, were enhanced by probiotics [123]. This increases intestinal integrity and prevents pathogen invasion. Owing to the fact that *C. cluster IV* and *C. cluster XIVa* are both Gram-positive bacterium and are primarily butyric acid producers in the gut, *C. cluster XIVa* can bind to mucoprotein, thus reducing the utilization of mucoprotein by intestinal pathogenic bacteria. *Lactobacillus* and *Bifidobacterium* can boost the cellular immune function and resistance of the host to pathogen-induced diarrhea, because it aids the proliferation of anaerobic Gram-positive bacteria [177].

*Firmicutes* and *Bacteroidetes* are important gut microbiota in broilers that function in energy production and metabolism, specifically in microbial fermentation and starch digestion [90] *Bacteroidetes* are involved in the formation of potential toxins via putrefaction that leads to the increases in pH of the intestinal contents, which is of benefit to gut health against acid-sensitive pathogens [178]. Some members of the Gram-negative *Bacteroidetes* phylum are known for their ability to degrade high-molecular-weight compounds such as carbohydrates and proteins, thereby supporting the host in acquiring more nutrients [150]. The ratio of *Firmicutes* to *Bacteroidetes* is critical to enhanced animal physiology and nutrient utilization of the host. The high *Firmicutes*/*Bacteroidetes* ratio in the ileum of NE-challenged birds could be due to the potential of probiotics to suppress the *C. perfringens* population and restore intestinal homeostasis; this enhances compensatory growth, which explains the improvement in FCR after NE challenge. The study of [13] revealed that probiotics enhanced the abundance of non-pathogenic *Clostridia* which belongs to phyla firmicutes and had a positive effect on gut integrity. This could be explained by the fact that the abundance of these microbes reduced the adherence of pathogens to the intestinal epithelium and toxin accumulation, because they contain the *cpa* gene that encodes the alpha toxin, one of the key toxins of CP. Probiotics increased *Actinobacteria* phyla [13]; this was a positive effect because one of the orders in *Actinobacteria* phyla is *Bifidobacteriales*, and *Bifidobacterium*-based probiotics have been proven to be effective against subclinical NE. *Lachnospiraceae*_UCG_010 was increased in NE-challenges birds due to probiotics [19]; the increased abundance may have explained the enhanced barrier function and reduced lesion scores. This because these spp are high in healthy individuals. *Clostridiales_vadinBB60* was enhanced by probiotics [19]. These are beneficial to intestinal functions because *Clostridiales_vadinBB60*_contains a variety of bacteria producing butyric acid. One study reported that an abundance of these spp is linked with increased serum antioxidant capacity [179]. The increase in the abundance of *Ruminococcaceae*, such as *Faecalibacterium prausnitzii*, is of positive significance because they are linked to the degredation of complex plant materials, which would in turn aid nutrient utilization. *Ruminococcus* spp. could produce lantibiotics that enhanced sterilization activity against some *Clostridia* and *Bifidobacteria* species [180]. Probiotics strains such as *B. pullicaecorum* restored intestinal microbiota after NE challenge [121], probably because *B. pullicaecorum* belongs to the *Ruminococcaceae* family, which harbors oxygen-sensitive butyrate-producing species which are critical to restoring microbiota balance. The higher abundance of lactobacillus [53], *L. salivarius* and *Bifidobacterium* [52], *Lactobacillus* and *Bacteroides* [121], *L. johnsonii* and *salivarius* [113] was notable due to probiotics supplementation in the NE-challenged birds. The abundance of *Lactobacillus* species in the gut is of positive significance and increased the recovery rate due to the array of positive attributes of lactobacillus species. *Lactobacillus* suppresses intestinal dysbiosis and maintains gut integrity owing to its various protective mechanisms: competitive exclusion, which prevents the adherence of pathogens to mucosal surfaces; lactic acid, disrupts the cell membrane which culminates in deleterious effects such as the inhibition of enzymatic activities; and the alteration of DNA structure and cell death [181]. *Lactobacillus* can form biofilms which can be used as barriers against enteropathogens, thus providing a conducive environment for gut cells’ proliferation and renewal [182]. *Lactobacillus* can produce bacteriocins against *Salmonella enteric* ATCC 25566, *Yersinia enterocolitica* ATCC 2371 and *Bacillus cereus* ATCC 49064, which could explain its antimicrobial activity [46]. *Lactobacilli* can produce antimicrobial substances such as hydrogen peroxide, organic acids and bacteriocins that act synergistically to suppress the proliferation of enteric pathogens in vivo [183]. *Lactobacillus* could degrade alpha toxins and reduce the synthesis of alpha toxins by CP [184]. The *L. casei* strain, as a safe probiotic bacterium, expresses NetB protein, thus making it a safe vaccine candidate against NE. In the study of [185], probiotics enhanced the serum anti-NetB antibody responses to NetB protein, and the resultant effect of better weight gain was notable. Synbiotics (PoultryStar me) consisting of various probiotic strains were found to enhance anti-CP IgA and decrease CP load in the guts of birds exposed to NE [137]. The direct effects of LAB include immunomodulation via attachment and interaction with enterocytes; antagonistic activity against pathogens by the production of lactate, thus lowering the pH and making the gastrointestinal tract environment unsuitable for acid-sensitive pathogens; and competitive exclusion mechanisms along within the production of bacteriostatic and bactericidal substances.

Probiotics’ modulation of gut microbiota may entail a reduction in the abundance of pathogenic phyla. *Proteobacteria* contain a wide variety of pathogens such as *Escherichia coli*, *Salmonella* and *Shigella*, which can colonize in the intestines of chickens. The reduction in *Proteobacteria* and *Bacteroidetes* in challenged birds suggests recovery. The increase in some species of *Bacteroidetes* was associated with a decrease in nutrient absorption [186]. *Clostridium. sensu stricto 1* proliferation was reduced with probiotics [53]. In birds fed an *L. fermentum*-supplemented diet, the abundance of *Romboutsia* spp. in the challenged birds was low [33]. *Romboutsia* spp. was reported to be associated with less severe immune responses accompanied with decreasing levels of pro-inflammatory cytokines in plasma [146]. Thus, the alleviation of intestinal inflammation and maintenance of microbial homeostasis may explain the positive gut health during NE challenge. *B. pullicaecorum* significantly reduced the abundance of *Escherichia*/*Shigella*, *Barnesiella*, *Desulfovibrio* and *Campylobacter* and was of positive value to gut health [121]. These Gram-negative organisms tend to enhance the synthesis of lipopolysaccharides (LPSs), which are endotoxins that stimulate localized or systemic inflammation, resulting in attenuated growth performance. *B. subtilis* supplementation caused a significant reduction in the relative abundance of pathogen-harboring phylum of *Proteobacteria* [32]; this reduction would protect the host from pathogens’ multiplication, which would protect the overall intestinal health of the host. The reduction in *Lachnopiraceae* [113] and *Faecalibacterium* [33] by probiotics enhances the resistance of birds to NE challenge. As much as probiotics alleviate NE effects on microbial shifts, Huang et al. [26] reported that CB could not effectively alter the microbiota composition after NE challenge. The aforementioned studies examined depicts the potentials of probiotics to modulate gut microbiota during NE challenge, which in turn provides conducive gut environment for symbiotic microflora; A healthy intestinal microbiota flora is crucial to overall health and physiological response of the host, due to its significant implications for immunity, inflammation, energy metabolism, nutrient availability and absorption rate, and productivity in broiler chickens.

### 4.8. Regulation of Metabolite Synthesis and Nutrient Transporters

The production of SCFAs in the gut, mainly acetate, propionate and butyrate, could be attributable to the fact that about 20% of intestinal microbiota functional genes are linked to carbohydrate metabolism [187]. Probiotics increased butyric acid, which would provide nutrients for villi development [32], thus contributing to the protective effect of probiotics on NE challenge. Membrane transport; carbohydrate metabolism; amino acid metabolism, replication and repair; and energy metabolism were the dominant functions of microbiota, which was confirmed by [188]. Membrane transport pathways are essential to cell viability and growth and are thereby crucial for the survival of bacteria in the gut ecosystem [189]. Decreased carbohydrate metabolism and increased amino acid metabolism were observed in the inflamed mucosal microbiota of ulcerative colitis patients [190]. *C. perfringens* challenge increased the amino acid metabolism of ileal microbiota but caused its reduction in the cecal microbiota as the disease progresses, which was conversely changed by probiotic addition [33]. Supplementation of probiotics enriched the predicted metabolism of butanoate and propanoate in the ileal microbiota compared to the negative control group [53]. This might be due to increased relative abundance of *Firmicutes* in the dietary group compared to other groups, as most butyrate producers belong to the *Firmicutes* phylum. Optimal butyrate production relies on the presence of butyrate-producing bacteria and various others including lactate-producing bacteria that cross-feed butyrate producers [191]. Butyrate could enhance epithelial regeneration by stimulating villus growth, absorption of butyrate and propionate by chicken cecal mucosa improve host energy metabolism and improve performance [192]. Carbohydrate metabolism were enriched in birds fed probiotics although under NE challenge, the high relative abundance of *Lactobacillus* spp. and *Bifidobacterium* in the probiotics supplemented group could account for the enriched pathway which benefits the energy of the host [52]. Also, high density of commensals bacteria in the gut can also hydrolyze indigestible carbohydrate of polysaccharides, oligosaccharides, and disaccharides to their compositional sugars, which gut bacteria ferment to produce short chain fatty acids that the host can use as energy. It could be inferred that beneficial microbes would proliferate and thrive; if gut microbiota replication and repair pathways are increased, and carbohydrates metabolized into SCFAs by gut microbes, which all culminate in improved intestinal function.

Taken together, the biological role of probiotics in the modification of intestinal pH, bacterial population, improvement of nutrient absorption and increased efficiency of feed utilization are linked to various underlying mechanisms. Notably, maintenance of healthy intestinal microflora, which aids intestinal integrity and promotes nutrient metabolism, pathogen exclusion principle would reduce the vulnerability of the host to pathogen, thereby reducing intestinal inflammation, stimulation of endogenous enzymes which improve the bioavailability of nutrients, gut fermentation which aids synthesis of SCFAs that maintain gut pH, supply energy to the host and nourish enterocytes for villi development. Improved growth performance evidenced by increased weight gain and reduced FCR, which may be attributable to increased feed consumption and nutrient digestibility, is reliant on gut health. Therefore, probiotics holds a lot of potentials as gut enhancers to improve growth performance and reduce mortality rate, which potentiates the economic benefit of broiler production.

## 5. Conclusions

Gut health is a key indicator of animal health and nutritional interventions may be used to enhance it. In the era of non-antibiotic use, probiotics which are natural feed additives are used as gut enhancers in poultry nutrition for birds under conventional poultry environment and disease challenge conditions such as necrotic enteritis. Probiotics could promote gut health through various mechanisms; modulation of intestinal microbiota structure, maintenance of intestinal integrity, nourishment of enterocytes for villi development, and regulation of immune and inflammatory response. All in all, probiotic effects on intestinal mucosa include maintenance, improvement, alleviation, control, and infection prevention.

## Figures and Tables

**Table 1:** Influence of probiotics on gut health of broilers under conventional poultry environment.

S/N	Probiotic Strain	GP	VM	IMF	INF	TJs	ANT	GM	Reference
1	*Bacillus coagulans*	NS	NE	P	P	NE	P	P	[15]
2	*Lactobacillus plantarum* ZLP001	NE	NE	P	P	P	NE	P	[24]
3	*Lactobacillus plantarum* A37 and *L. plantarum* MIII	P	P	P	P	P	P	P	[36]
4	*Bacillus subtilis* and *Bacillus licheniformis*	P	P	P	NE	NE	P	NE	[37]
5	*Lactobacillus* LP184 and *Yeast* SC167	P	P	P	NE	NE	P	P	[38]
6	*Bacillus amyloliquefaciens* TL106	P	P	P	P	P	NE	P	[39]
7	*Lactobacillus paracaesi*	NS	P	P	P	P	P	P	[40]
8	*Multi strain*	P	P	P	NE	P	P	NE	[41]
9	*Bacillus amyloliquefaciens*	P	NE	NE	P	P	NE	NE	[42]
10	*Bacillus subtilis*	P	P	P	P	P	P	NE	[43]
11	*Bacillus methylotrophicus* SY200	P	P	NE	NE	P	P	P	[44]
12	*Bacillus subtilis* and *Bacillus licheniformis*	P	NE	P	P	NE	P	P	[45]
13	*Lactococcus* and *Lactobacillus plantarum*	P	P	P	NE	NE	NE	P	[46]
14	*Bacillus subtilis fmbj*	P	P	P	NE	NE	P	NE	[47]
15	*Bacillus subtilis*	P	P	P	NE	P	NE	P	[54]
16	*Bacillus subtilis (B. subtilis)* BYS2	P	P	P	P	NE	NE	NE	[55]
17	*Clostridium butyricum*	P	P	NE	P	P	NE	P	[56]
18	*Bacillus* spp. and *Debaryomyces* spp.	P	P	NS	NE	NE	P	P	[57]
19	*Saccharomyces cerevisiae hydrolysate*	P	P	NE	P	P	NE	P	[58]
20	*Bacillus subtilis* DSM 32315	P	P	NS	NS	NE	NE	P	[59]
21	*Bacillus subtilis* ATCC19659	P	P	P	NE	NE	P	P	[60]
22	*Lactobacillus* and *Yeast*	P	P	NE	NE	NE	P	NS	[61]
23	*Bacillus amyloliquefaciens* LFB112	P	NE	P	NE	NE	NE	NE	[62]
24	*Bacillus* spp.	P	P	NE	NE	NE	NE	P	[63]
25	*Bacillus amyloliquefaciens* CECT 5940	P	NE	P	P	NE	P	NE	[64]
26	*Lactobacillus*	P	NE	P	NS	NE	NS	P	[65]
27	*Bacillus subtilis*	P	P	NE	NE	NE	NE	NE	[66]
28	*Bacillus amyloliquefaciens* SCO6	NS	NE	P	P	P	P	P	[67]
29	*Lactobacillus*	NS	NE	P	NE	P	NE	NE	[68]
30	*Lactobacillus reuteri*	NS	NE	NE	NE	NE	NE	P	[69]

NS—non-significant; NE—not evaluated; P—positive effect; GP—growth performance; VM—villi morphology; IMF—immune function; INF—intestinal inflammation; TJs—tight junction proteins; ANTC, antioxidant capacity; GM—gut microbiota.

**Table 2:** Influence of NE challenge on induction of gut lesions, mortality rate, growth performance and the ameliorative effect of probiotics.

	Probiotic Strain	Response to NE Challenge			Response to Dietary Probiotics			Ref
S/N		Lesion Site	Mortality Rate	GP	Lesion Site	Mortality Rate	GP	
1	*B. amyloliquefaciens* CECT 5940	Jejunum	Comparable to positive control	Reduced growth performance	No effect	Not significant	Improved performance	[11]
2	*B. licheniformis* H2	NE	High mortality rate	Reduced growth performance	NE	Reduced mortality rate	Enhanced weight gain and reduced FCR	[12]
3	Multi strains of *Lactobacillus*	Small intestine	High mortality rate	Reduced growth performance	Reduced lesion scores	Reduced mortality rate	Enhanced weight gain and reduced FCR	[13]
4	*B. subtilis* DSM29784	Comparable to control	High mortality rate	Reduced growth performance	NE	Reduced mortality rate	Enhanced weight gain	[17]
5	*B. subtilis*	Jejunum and ileum	High mortality rate	Reduced growth performance	Reduced lesion scores	Reduced mortality rate	Reduced FCR	[18]
6	*B. licheniformis*	Duodenum, jejunum and ileum	High mortality rate	Reduced growth performance	Reduced lesion scores	Reduced mortality rate	Reduced FCR	[19]
7	*Bacillus*	Ileum	High mortality rate	Reduced growth performance	Reduced lesion scores	Reduced mortality rate	Improved weight	[21]
8	*B. licheniformis*	Duodenum, jejunum and ileum	High mortality rate	Reduced growth performance	Reduced lesion scores	Reduced mortality rate	Reduced FCR	[23]
9	*B. coagulans*	Duodenum and jejunum	High mortality rate	Reduced growth performance	Reduced lesion scores	Reduced mortality rate	Enhanced weight gain and reduced FCR	[25]
10	*Clostridium. butyricum*	Intestine	High mortality rate	Reduced growth performance	Reduced lesion scores	Reduced mortality rate	Reduced FCR	[26]
11	*L. plantarum* 1.2567	Small intestine	High mortality rate	Reduced growth performance	Reduced lesion scores	Reduced mortality rate	Improved weight gain	[30]
12	*B. amyloliquefaciens* H57.	Small intestine	High mortality rate	Reduced growth performance	Reduced lesion scores	Reduced mortality rate	Enhanced weight gain and reduced FCR	[31]
13	*B. subtilis*	Duodenum, jejunum and ileum	High mortality rate	Reduced growth performance	Reduced lesion scores	Reduced mortality rate	Reduced FCR	[32]
14	*L. fermentum, B. coagulans*	Duodenum and Ileum	High mortality rate	Reduced growth performance	no effect	Reduced mortality rate	Reduced FCR	[33]
15	*B. subtilis* DSM 32315.	NE	High mortality rate	Reduced growth performance	NE	Reduced mortality rate		[52]
16	Multi strain	Duodenum, jejunum and ileum	High mortality rate	Reduced growth performance	Reduced lesion scores	Reduced mortality rate	Reduced FCR	[53]
17	*Lactobacillus johnsonii* BS15	NE	High mortality rate	Reduced growth performance	NE	Reduced mortality rate	Enhanced weight gain and reduced FCR	[70]
18	Primlac: multi strain	Duodenum and jejunum	High mortality rate	Reduced growth performance	Reduced lesion scores	Reduced mortality rate	Reduced FCR	[94]
19	*B. subtilis* DSM29784	Jejunum	High mortality rate	Reduced growth performance	Comparable to control	Reduced mortality rate	Enhanced weight gain and reduced FCR	[95]
20	*B. licheniformis* H2	Ileum	High mortality rate	Reduced growth performance	NO	Reduced mortality rate	Enhanced weight gain and reduced FCR	[109]
21	*B. amyloliquefaciens* BLCC1-0238	Duodenum	High mortality rate	Reduced growth performance	Reduced lesion scores	Reduced mortality rate	Enhanced weight gain and reduced FCR	[110]
22	Multi strain	Duodenum, jejunum and ileum	High mortality rate	Reduced growth performance	Reduced lesion scores	Reduced mortality rate	Enhanced weight gain and reduced FCR	[111]
23	*E. faecium*	Jejunum	High mortality rate	Reduced growth performance	Reduced lesion scores	Reduced mortality rate	Enhanced weight gain and reduced FCR	[112]
24	*B. subtilis* DSM 32315	Footpad	High mortality rate	Reduced growth performance	Reduced lesion scores	Reduced mortality rate	Enhanced weight gain and reduced FCR	[113]
25	*L. johnsonii* BS15	NE	High mortality rate	Reduced growth performance	NE	Reduced mortality rate	Enhanced weight gain and reduced FCR	[114]
26	*C. butyricum* MIYAIRI 588	Small intestine	High mortality rate	Reduced growth performance	Reduced lesion scores	Reduced mortality rate	Enhanced weight gain and reduced FCR	[115]
27	*L. johnsonii* BS15, Bacillus	NE	High mortality rate	Reduced growth performance	NE	Reduced mortality rate	Enhanced weight gain and reduced FCR	[116]
28	*B. subtilis* DSM 32315	Mid intestine	High mortality rate	Reduced growth performance	Reduced lesion scores	Reduced mortality rate	Enhanced weight gain and reduced FCR	[117]
29	Bacteriophage	Jejunum	High mortality rate	Reduced growth performance	Reduced lesion scores	Zero mortality in high-dose PRO group	Improved weight gain	[118]
30	*Bacillus.* Surfactin (fermented product)	Duodenum, jejunum and ileum	High mortality rate	Reduced growth performance	Reduced lesion scores	Reduced mortality rate	Enhanced weight gain and reduced FCR	[119]
31	*B. licheniformis*	Duodenum and jejunum	High mortality rate	Reduced growth performance	Reduced lesion scores	Reduced mortality rate	Enhanced weight gain and reduced FCR	[120]
32	*Butyricicoccus pullicaecorum* 25-3T	Duodenum and jejunum	High mortality rate	Reduced growth performance	Reduced lesion scores	Reduced mortality rate	Reduced FCR	[121]
33	*B. subtilis* DSM29784	Ileum	Low mortality rate	Reduced growth performance	Reduced lesion scores	Reduced mortality rate	No effect	[122]
34	*L. johnsonii*. LB 15	NE	High mortality rate	Reduced growth performance	NE	Reduced mortality rate	Enhanced weight gain and reduced FCR	[123]
35	*B. amyloliquefaciens* CECT 5940	Footpad	Comparable to positive control	Reduced growth performance	Reduced lesion scores	Reduced mortality rate	Enhanced weight gain and reduced FCR	[124]

NE-not evaluated, FCR-feed conversion ratio.

**Table 3:** Influence of NE challenge on intestinal barrier function and modulation effects by probiotics.

S/N	Response to NE Challenge	Probiotic Strain	Response to Dietary Probiotics	Ref
1	Decreased the expression of CLDN-1, CLDN-3, ZO-1 and ZO-2	*B. licheniformis* H2	Increased the expression of these proteins	[12]
2	Reduced the expression of IGF-1 and EGF in the gut	Multi probiotic strain. *B. subtilis*	Increased IGF-1 and EGF in the jejunum and ileum	[18]
3	Increased expression of CLDN3,	Multi strain	Decreased CLDN3; increased CLDN3 and Muc-2 on day 42	[19]
4	Reduced the expression of CLDN-3	*B. licheniformis*	Increased the expression of CLDN-3	[23]
5	Reduced mRNA expression of Muc2; reduced IAP activity	*B. coagulans*	No effect on mucin expression; increased IAP activity in the jejunum	[25]
6	Decreased CLDN-1 but had no influence on OCLDN and CLDN-2	*L. fermentum*, *B. coagulans*	Increased CLDN-3	[26]
7	Reduced mRNA expression of CLDN-3, CLDN-1 and ZO-2	Primlac: multi strain probiotics	Increased expression of these proteins	[94]
8	Reduced expression of CLDN-1 and OCLDN transcripts; increased Muc-2	*B. subtilis* DSM29784	No effect on expression of claudins	[95]
9	Increased level of serum DAO and reduced CLDN-3 and MUC-2	*B. amyloliquefaciens* BLCC1-0238	Reduced level of serum DAO and D-lactic acid; increased OCLN, ZO-1 and MUC2	[110]
10	Reduced CLDN-3 and ZO-1; increased MLCK mRNA expression	*E. faecium*	Increased expression of CLDN-1	[112]
11	Reduced JAM2	*B. licheniformis*	Increased JAM2	[122]

**Table 4:** Influence of NE challenge on intestinal villi morphology and the regulatory effects of probiotics.

S/N	Response to NE Challenge	Probiotic Strains	Response to Dietary Probiotics	Ref
1	Hyperemia of lamina propria and necrotic intestinal epithelial cells	*B. licheniformis*	Restored it	[12]
2	Reduced V/C ratio	Multi strains of *Lactobacillus*	Increased V/C ratio	[13]
3	Decreased goblet number and no effect on jejunal VH, CD and V/C ratio	Multi strain	No significant effect on VH, CD and V/CD	[19]
4	Reduced villi length, necrosis of intestinal villi and hyperplasia	*B. subtilis*	Restored villi morphology	[22]
5	Reduced VH/CD, goblet cell number, VH and IAP activity	*B. coagulans*	Restored the increased negative effect	[25]
6	Damaged ileal tissue, loss of villi architecture, mucosal damage and decreased density and length of villi enterocytes	*L. plantarum* 1.2567 powder	Restored it and reduced NO and MPO activity in the ileum mucosa	[30]
7	Irregular villi, oedma, separation from basement membrane and goblet cell metaplasia	*B. subtilis*	Restored the villi architecture, and reduced oedema	[32]
8	Irregular villi structure and shorter jejunal villi length	*B. subtilis* DSM29784	Restored it	[95]
9	Reduced villi length and VH/CD	*B. licheniformis*	Restored it	[109]
10	Irregular villi shape, swelling of villus tip and reduced villi length	*B. amyloliquefaciens*, BLCC1-0238	Restored it	[110]
11	Increased intestinal histopathology	*E. faecium*	Increased PCNA-positive cells and reduced TUNEL -positive cells	[112]
12	Damaged villi, shedding of epithelial cells and congested lamina propria	*L. johnsonii* BS15	Restored it	[114]
13	Reduced villi length	*L. johnsonii, Bacillus*	Restored it	[116]
14	Increased cecal mucosal thickness	*B. subtilis* DSM 32315	Decreased CD, TLI and EI; increased V/C ratio	[117]
15	Disruption in villi crypt and lamina propria; reduced villi length	Bacteriophage	Restored it	[118]
16	Reduced villi length	*Bacillus*. Surfactin (fermented product)	Restored villi morphology	[119]
17	Reduced villi length	*B. licheniformis* fermented product	Restored villi morphology	[120]
18	Reduced villi length and increased duodenal crypt depth	*B. licheniformis*	Increased villi length and reduced crypt depth	[136]
19	Decreased jejunal height	Multi strain	Improved jejunal V/C ratio	[137]

**Table 5:** Intestinal immune and inflammatory response to influence of NE challenge and modulation effect by probiotics.

	Response to NE Challenge		Response to Dietary Probiotics			
S/N	Intestinal Immunity	Intestinal Inflammation	Probiotic Strains	Intestinal Immunity	Intestinal Inflammation	Ref
1	Reduced expression of IgA and IgG; increased PGC-1a expression	NE	*B. licheniformis* H2	Increased IgA and IgG; reduced expression of PGC-1a	NE	[12]
2	Decreased immunoglobulins and T helper: Th cells in the cecal tonsils	Increased IL-1B	Multi strains of *Lactobacillus*	Increased immunoglobulins and T cells. Reduced sIgA	Increased IFN-γ, IL-13 and IL-2; reduced IL-1B, IL-12p35, IL-17 and TGF-B	[13]
3	Decreased jejunal mRNA TRIF and NF-KB	Changes in IL-1β, IL-10, IL-17 and TNF-α	*B. licheniformis*	Increased jejunal mRNA TRIF and NF-KB; no effect on TLR2 or TLR4	No effect on IL-1β, IL-10, IL-17 or TNF-α; increased GFs and HSP proteins	[19]
4	Increased FITC and intestinal IgA	NE	*B. licheniformis* H2	Increased the contents	NE	[21]
5	Increased sIgA	Increased IFN-γ	*B. licheniformis*	Reduced sIgA	Reduced IFN-γ, IL-10 and IL-17	[23]
6	Reduced sIgA, TLR2, TLR4 and TNFSF15 Fowlcidin gene	Increased IFN-γ. Decreased TLR2, TLR4 and TNFSF15 gene	*B. coagulans*	Increased sIgA; no effect on TLRsl only increased fowlcidin-2	Reduced IFN-γ; no effect on others	[25]
7	Reduced TLR2	No effect on TNF or TLR2; increased IL-17	*C. butyricum*	No effect on intestinal IgA	Increased TNF-α, IL-10, reduced IL-17A	[26]
8	NE	Increased (chTNF-α) and IL-1β in the ileum mucosa	*L. plantarum* 1.2567	NE	Reduced (chTNF-α) and IL-1β	[30]
9	Reduced populationof CD3+	Increased jejunal IL-1β and TGF-β4 by 28. Both increased	*L. fermentum*, *B. coagulans*	Increased CD3	Increased and reduced IL-1β, INF-γ, IL-13,1L-17 and TGF-B.	[33]
10	NE	Increased IL-6, TNF-a and IFN-γ	*B. subtilis* DSM 32315.	NE	Reduced IL-6, TNF-α and IFN-γ and increased IL-10 and SIgA	[52]
11	Reduced ileum IgA and IgG, sIgA and content. Increased MMP-2	Increased IFN-γ and IL-10	*Lactobacillus johnsonii* BS15	Increased the IgA, IgG and sIgA content; reduced MMP-2	Reduced IFN-γ and IL-10; increased Nrf-2 and IL-8.	[70]
12	NE	Reduced IL-10 and IL-17	Primlac:multi strain	NE	Increased IL-10 and IL-17	[94]
13	Reduced serum sIgA and IgG	Increased IL-1β, TNF-α, INF-γ and IL-6	*B. subtilis* DSM29784	No effect on sIgA	Reduced IFN-γ and TNF- α	[95]
14	Reduced sIgA	Increased IFN-γ, IL-10 and IL-6	*B. amyloliquefaciens* BLCC1-0238	Increased sIgA	Decreased IFN-γ, IL-10 and IL-6	[110]
15	NE	Reduced TLR-2, IL-1β, IL-4, IFN-γ, iNOS and IL-10	*E. faecium*		Increased MYD88, NFK-B, IL-1β, IL-4 and iNOS	[112]
16	Reduced serum IgG and IgA, CD3+, CD4+ and lymphocyte percentage	Reduced IL-2, IL-4 and IFN-γ	*L. johnsonii* BS15	Increased the IgG and IgA and CD+ cells	Increased IFN-γ and IL-2	[114]
17	Reduced serum IgG and IgM	No effect on IL-6, TNF-α or IFN-γ	*L. johnsonii* BS15, *Bacillus*	Increased serum IgG and IgM	No effect on IL-6, TNF-α or IFN-γ but reduced IL-8	[116]
18	Reduced TLR21	Reduced INF-γ, IL-12 and TGF-B4	*B. subtilis* 29,784	Increased TLR21 after infection and TLR 5	Increased INF-γ, IL-12 and TGF-B4	[122]

**Table 6:** Influence of NE challenge on gut microbiota and the modulation effect by probiotics.

S/N	Response to NE Challenge	Probiotic Strain	Response to Dietary Probiotics	Ref
1	Decreased *Actinobacteria*, *Lactobacillacae* and *Firmicutes* (*Clostridia*)	Multi strains of *Lactobacillus*	Increased *Actinobacteria*, *Lactobacillacae* and *Firmicutes* (*Clostridia*)	[13]
2	Decreased *Lachnospiraceae_*UCG_010, *Clostridiales*_vadinBB60 and *Ruminococcaceae*_NK4A214_	*B. licheniformis*	Increased *Lachnospiraceae_*UCG_010, *Clostridiales_*vadinBB60 and *Ruminococcaceae_*NK4A214	[19]
3	Reduced *Firmicutes*, increased genera; *Turicibacter*, *Streptococcus*, *Enterococcus* and *Clostridium*	*B. licheniformis* H2	Reduced *Proteobacteria*, increased *Lactobacillus* and *Bacillus*	[21]
4	Increased *Clostridium sensu stricto-*1 and reduced *Lactobacillus*	*B. licheniformis*	Reduced *Clostridium sensu stricto 1* and *Escherichia-Shigella*; increased *Lactobacillus*	[23]
5	Reduced cecal *Lactobacillus* and *Bifidobacterium*; increased cecal *coliform*	*B. coagulans*	Reversed the trend	[25]
6	Increased *Candidatus Arthromitus* unclassified *Brachybacterium* and decreased *Lactobacillus* sp. KC45b	*C. butyricum*	Probiotics reversed all; increased *Weissella thailandensis* and *Pediococcus acidilactici*	[26]
7	Increased *Dorea*, *Bacteroides*, *Eubacterium*, *Caldanaerocella* and *Enterococcus*	*B. subtilis*	Decreased *Dorea, Ruminococcus* and *Proteobacteria*	[32]
8	Increased *Romboutsia*, *f_Lachnospiraceae* and *Ruminococcus_torques* group, and decreased *Lactobacillus*. Lower ileal *Bacteriodetes* and cecal *Proteobacteria* on day 28	*L. fermentum, B. coagulans*	Probiotics reversed it; decreased *Faecalibacterium* spp. and *f_Peptostreptococcaceae*	[33]
9	Increased proliferation of CP; reduced *L. salivarius* and *B. fidobacterium*	*Bacillus subtilis* DSM 32315.	Reduced proliferation of CP; increased *L. Salivarius* and *B. fidobacterium*	[52]
10	Increased *Prevotellacea*, *Muribaculacea*, *Rominiclostridium* 9, *Oscillibacter*, RuminococcaceaeUCG_014, ASF356, *Clostridium sensu stricto 1*,	Multi strain	Decreased the abundance of the pathogens; increased ileum *Firmicutes* and *Lactobacillus*	[53]
11	Increased *Enterococcus*, *Escherichia*/*Shigella*, *Barnesiella*, *Desulfovibrio* and Campylobacter; reduced *Lactobacillus* and *Bacteriodes*	Primlac: multi strain probiotics	Reduced *Enterococcus, Escherichia*/*Shigella*, *Barnesiella*, *Desulfovibrio* and Campylobacter; increased *Lactobacillus* and *Bacteriodes*	[94]
12	Reduced *Ruminococcaceae* and *Bifidobacterium*	*B. subtilis* DSM29784	Increased *Ruminococcaceae* and *Bifidobacterium,*	[95]
13	Reduced *Lactobacillus*	*E. faecium*	Reversed the negative effect	[112]
14	Increased *lachnopiraceae* and *Ruminococcaceae*	*B. subtilis* DSM 32315	Increased *L. johnsonii* and *Salivarius*; reduced CP alpha toxin	[113]
15	Increased CP coliforms in the ileum and cecum	Bacteriophage	Reduced CP *coliforms* in the ileum and cecum	[118]
16	Increase in *Escherichia coli*, *Staphylococcus aureus*, *Salmonella typhimurium* and *C. perfringens*.	*Bacillus*. Surfactin (fermented product)	In vitro reduction in *Escherichia coli*, *Staphylococcus aureus*, *Salmonella typhimurium* and *C. perfringens*	[119]
17	NE reduced *Faecalibacterium*	*B. subtilis* 29784	Increased *Butyricicoccus* and *Faecalibacterium* genera	[122]
18	Reduced *Bacteriodetes*, *C. cluster IV* and *C. cluster XIVa*, *Lactobacillus*, increased *Streptococcus* spp. *Enterobacteriaceae*	*Lactobacillus johnsonii*. LB 15	Increased *Lactobacillus*, *C. cluster* IV and *C. cluster XIVa*, reduced *Streptococcus* and *Enterobacteriaceae*	[123]
